# Neurotrophic and Neuroprotective Actions of (−)- and (+)-Phenserine, Candidate Drugs for Alzheimer’s Disease

**DOI:** 10.1371/journal.pone.0054887

**Published:** 2013-01-30

**Authors:** Anna M. Lilja, Yu Luo, Qian-sheng Yu, Jennie Röjdner, Yazhou Li, Ann M. Marini, Amelia Marutle, Agneta Nordberg, Nigel H. Greig

**Affiliations:** 1 Alzheimer Neurobiology Center, Department of Neurobiology, Care Sciences and Society, Karolinska Institutet, Karolinska University Hospital Huddinge, Stockholm, Sweden; 2 Drug Design and Development Section, Laboratory of Neurosciences, Intramural Research Program, National Institute on Aging, National Institutes of Health, Baltimore, Maryland, United States of America; 3 Department of Neurological Surgery, Case Western Reserve University School of Medicine, Cleveland, Ohio, United States of America; 4 Department of Geriatric Medicine, Karolinska University Hospital Huddinge, Stockholm, Sweden; 5 Department of Neurology and Neuroscience Program, Uniformed Services University of the Health Sciences, Bethesda, Maryland, United States of America; University of Ulster, United Kingdom

## Abstract

Neuronal dysfunction and demise together with a reduction in neurogenesis are cardinal features of Alzheimer’s disease (AD) induced by a combination of oxidative stress, toxic amyloid-β peptide (Aβ) and a loss of trophic factor support. Amelioration of these was assessed with the Aβ lowering AD experimental drugs (+)-phenserine and (−)-phenserine in neuronal cultures, and actions in mice were evaluated with (+)-phenserine. Both experimental drugs together with the metabolite N1-norphenserine induced neurotrophic actions in human SH-SY5Y cells that were mediated by the protein kinase C (PKC) and extracellular signal–regulated kinases (ERK) pathways, were evident in cells expressing amyloid precursor protein Swedish mutation (APP_SWE_), and retained in the presence of Aβ and oxidative stress challenge. (+)-Phenserine, together with its (−) enantiomer as well as its N1- and N8-norphenserine and N1,N8-bisnorphenserine metabolites, likewise provided neuroprotective activity against oxidative stress and glutamate toxicity via the PKC and ERK pathways. These neurotrophic and neuroprotective actions were evident in primary cultures of subventricular zone (SVZ) neural progenitor cells, whose neurosphere size and survival were augmented by (+)-phenserine. Translation of these effects *in vivo* was assessed in wild type and AD APPswe transgenic (Tg2576) mice by doublecortin (DCX) immunohistochemical analysis of neurogenesis in the SVZ, which was significantly elevated by 16 day systemic (+)-phenserine treatment, in the presence of a (+)-phenserine-induced elevation in brain- derived neurotrophic factor (BDNF).

## Introduction

Alzheimer’s disease (AD), a progressive neurodegenerative condition resulting in memory loss and neuropsychiatric disturbances, is the most common form of dementia and afflicts in excess of 26 million people worldwide [1]. Its economic burden and impact on the quality of life of both patients and their caregivers are overwhelming and continue to escalate [2], [3]. Existing therapeutic options remain limited, and numerous recent AD experimental drugs have failed to demonstrate clinical efficacy [4]. The concerning 66% rise in the proportion of AD related deaths that occurred between 2000 and 2008, in the face of declines in deaths from the leading illnesses of heart disease, stroke, and prostate cancer [5], highlight the fact that whereas currently available drugs may ameliorate disease symptoms, they do not appear to impact disease progression. Hence, new effective AD treatment strategies are greatly needed [6].

Key hallmarks of AD are the appearance within the brain of misfolded protein aggregates that form senile plaques and neurofibrillary tangles (NFTs). The former largely comprise of amyloid-β peptide (Aβ), a self-aggregating peptide that forms toxic oligomers [7] and that derives from the proteolytic processing of amyloid precursor protein (APP), an integral membrane protein present on many cells and concentrated in the synapses of neurons [8], [9]. NFTs are formed from hyperphosphorylation of the microtubule-associated protein, tau. The development of these pathological changes leads to oxidative stress and excitotoxicity, synaptic loss, particularly of cholinergic neurons, changes in brain neurotrophin levels essential to support neuronal survival and neurogenesis, progressive cellular dysfunction and, eventually, neuronal death [7], [8], [10], [11]. Each of these features provides a potential target to treat AD.

In large part, age and disease related declines in brain neurotrophin levels that are critical in supporting and maintaining the postnatal neuronal architecture, particularly that of the hippocampus, can unfavorably alter the balance between neuronal survival and demise and lead to neurodegeneration [12], [13], [14]. Neurotrophins, additionally, promote neural stem cell proliferation, differentiation and survival of resulting neurons and glia, and can thereby influence learning, memory and behavior [15], [16], [17].

The two separate and chirally pure experimental AD drugs (−)- and (+)-phenserine appear to ameliorate a number of cardinal features of AD. (−)-Phenserine, which reached phase 3 clinical trials [18] and is currently being reformulated to optimize its pharmacological actions [19], and (+)-phenserine, which has recently undergone phase 1 tolerability and target engagement trials [20], are both APP synthesis inhibitors and, thereby, lower Aβ levels [21], [22], [23], [24]. (−)-Phenserine furthermore ameliorates the cholinergic deficiency prominent in AD via its acetylcholinesterase inhibitory actions [21] and, thereby, augments cognition [18], [25]. Additionally, (+)-phenserine augments neurogenesis [26], [27] and lowers both total and phosphorylated tau levels *in vivo* [20]. In light of the decline in neurotrophins in AD brain, we characterized the actions of both (−)- and (+)-phenserine as neurotrophic and neuroprotective agents in both neural stem cell and adult neuronal cultures. Both agents, together with specific metabolites, demonstrated efficacy at pharmacologically achievable concentrations, which translated to wild type (wt) and in an AD transgenic mouse model (Tg2576).

## Materials and Methods

### Ethics Statement

All animal experimental procedures were carried out in compliance with guidelines for the National Institutes of Health for Care and Use of Laboratory Animals, the Swedish National Board for Laboratory Animals, and were approved by the Animal Research Committee (s43/07 and s53/10), Karolinska Institutet, Stockholm, Sweden.

### Cell Culture

#### SH-SY5Y cells

Human neuroblastoma SH-SY5Y cells were obtained from American Type Culture Collection (ATCC, Manassa, VA, USA), and SH-SY5Y cells transfected with the Swedish double mutation K670N/M671L (APPswe) were a gift from Dr. Eirikur Benedikz, Karolinska Institutet, Sweden. Cells were cultured in Eagle’s minimum essential medium and Ham’s F12 medium containing fetal bovine serum (10%) and penicillin-streptomycin (1%) (Invitrogen, La Jolla, CA).

#### Subventricular zone cell culture

SVZ progenitor cells were isolated from the lateral and medial ganglionic eminence of wt or Tg2576 mouse embryos at embryonic day E13.5. The tissues were triturated to single cell suspension and plated in neurobasal medium without glutamine, and were supplemented with B27 (1∶50 dilution), 2 mM L-glutamine, 1% penicillin/streptomycin, 2 µg/ml heparin (Invitrogen, La Jolla, CA), epidermal growth factor (20 ng/ml, Imgenex) and basic fibroblast growth factor (10 ng/ml, R&D systems). Notably, small differences in the culture substratum may influence the formation of neurospheres, and we attempted to maintain similar conditions across our studies. These cells were exposed to 0.01–1 µM (+)-phenserine, inhibitors of PKC (GF109203X, 2.5 µM), MEK1/2 (U0126, 5 µM) or MEK1 (PD98059, 10 µM), or vehicle following plating at the density 10^4^ cells/cm^2^. The size and number of the formed neurospheres were quantified on day 6 or 7 *in vitro* (DIV6 or 7).

### Viability/cell Proliferation Assays

SH-SY5Y cells were plated at a density of 20,000 cells/100 µl in 96-well plates. After 24 hr, cells were exposed to increased concentrations (3–300 µM) of (+)-phenserine, and 30 µM concentration of (−)-phenserine, (+)-N1-norphenserine, (+)-N8-norphenserine and (+)-N1,N8-bisnorphenserine. Inhibitors of PKC (GF109203X, 2.5 µM), MEK1/2 (U0126, 5 µM) or MEK1 (PD98059, 10 µM) were added to the cells 30 min prior to addition of (+)-phenserine or vehicle. In parallel experiments, cells were exposed to (+)-phenserine 24 hr before addition of 0.1, 1 or 10 µM Aβ42 or 10, 30 or 100 µM H_2_O_2_, or 100 mM glutamate before measuring cell proliferation. After an additional 24 hr, MTS assays were performed using the CellTiter 96 Aqueous One Solution Cell Proliferation Assay kit (Promega, Madison, WI, USA) according to the manufacturer’s instructions.

### Animals

APPswe transgenic mice (Tg2576) were used in the present study. These C57B6JxSJLF1 mice overproduce APP 695 that carries the Swedish mutation (K670N/M671L), driven by a hamster gene promotor. The mice were inhouse bred at the Karolinska Institutet, Stockholm, Sweden and backcrossed to B6SJLF1 mice. Their genotype was determined with PCR [28] and wild type (wt) littermates served as control animals throughout the study. All animals were housed in enriched cages with *ad libitum* access to food and water during a 12/12 hr light/dark cycle. Embryos from pregnant Tg2576 transgenic mice were used for isolation of SVZ cells, as described previously [29].

### Drug Treatment

Mice at 4–6 months of age (Tg2576 (n = 16)) and age matched wt controls (n = 13) were administered either (+)-phenserine (25 mg/kg) or vehicle (0.9% saline) by intraperitioneal injection once a day for 16 consecutive days. The day after the last injection, all animals were anesthetized with a 1∶1 mixture of ketamine (100 mg/kg) and xylazine (20 mg/kg) and euthanized by transcardial perfusion with PBS. The brains were collected, one hemisphere was post-fixed with 4% paraformaldehyde (pH 7.4), and stored at −80°C for later use in immunohistochemical staining. The other hemisphere was stored at −80°C without post-fixation for subsequent BDNF ELISA measurements.

### Doublecortin Immunohistochemistry

Brain hemispheres were sectioned in the sagittal plane to allow doublecortin (DCX) immunohistochemical analysis of the SVZ. Sections were incubated overnight with goat anti-DCX (1∶500, Santa Cruz), and DCX was visualized with rabbit anti-goat streptavidin-horseradish peroxidase conjugate (1∶500, Vector Laboratories) and by using the ABC method with nickel-enhanced diaminobenzidine. DCX expression levels in the SVZ were quantified by measuring the intensity of DCX^+^ immunostaining. First, images of the lateral wall of the SVZ were captured in 4× magnification using a light microscope (Nikon, Tokyo, Japan). Software ImageJ (NIH, Bethesda, MA) was then used to measure the intensity in a specified region in triplicate sections per animal. The background intensity was measured and subtracted from the original values in each section, and mean intensities and standard error of the mean were calculated using GraphPad Prism version 5.0 (GraphPad Software, La Jolla, CA).

### BDNF ELISA

Cytosolic fractions were obtained from dissected cortical tissue of saline and (+)-phenserine treated mice (4–6 months). In brief, tissues were sonicated in 1∶50 g/ml buffer containing 150 mM NaCl, 1 mM EDTA, 1 mM DTT, 50 mM Tris·HCl, 1% Nonidet P-40, 10% glycerol, and a protease and phosphotase inhibitor mixture (Roche Applied Science). The homogenates were centrifuged at 8,000×*g* for 5 min at 4°C, and the supernatants were removed and stored at –80°C until use. Rabbit polyclonal antibody to rat brain-derived neurotrophic factor (BDNF) (Abcam) was biotin labeled according to instructions from the manufacturer (Roche). The labeled antibodies were collected and saved in 50% glycerol at −80°C until use. Rabbit polyclonal antibody to mouse anti-BDNF (1∶5000, Abcam), was used to coat the wells of 96-well microtiter plates. After incubation over night at 4°C by shaking, the wells were blocked with 5% bovine serum albumin in coating buffer and incubated for 2 hr at room temperature. The blocking buffer was discarded and the wells were washed three times with 300 µl TBS-T. As antigen, 100 µl brain extracts were added to the wells and incubated over night at 4°C. Unbound antigens were discarded and the wells were washed two times with 300 µl PBS. BDNF was detected using 100 µl biotin labelled Ab6201 antibody and incubated for 3 hr at room temperature. After washing, streptavidin (1∶5000 in 0.1% TBS-T-BSA) was added and the plate was incubated at room temperature for 2.5 hr. After four washes with TBS-T, 200 µl substrate (4-Nitrophenyl phosphate-Na_2_-6H_2_O), diluted in DEA buffer (1 M diethanolamine, 0.5 M MgCl_2_. 6H_2_O, 0.01% NaN_3_) was applied and the plate was incubated at room temperature for 3–5 hr in darkness. The optical density was read at 405 nm wavelength.

### Statistical Analyses

Data is expressed as mean values ± SEM. Statistical differences between treatment groups were determined with student’s t-test or One-way ANOVA followed by Bonnferroni’s or Dunnet’s *post hoc* tests.

## Results

### Neurotrophic Actions of (+)- and (−)-phenserine in SH-SY5Y Cell Cultures

**Figure 1:**
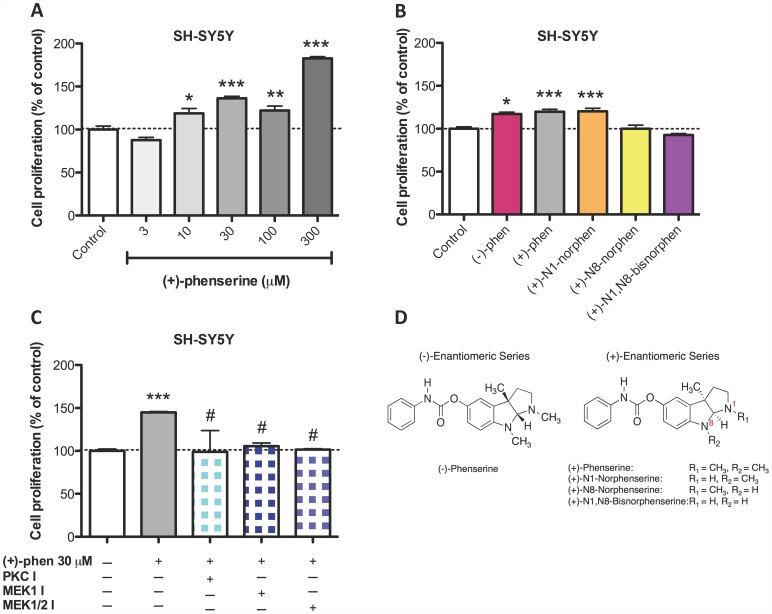
(+)- And (−)-phenserine induce neurotrophic actions, as assessed by increasing cellular proliferation of SH-SY5Y cells. SH-SY5Y cells were exposed for 24 hr to increased concentrations (3–300 µM) of (+)-phenserine (A), and 30 µM concentration of (−)- and (+)-phenserine, (+)-N1-norphenserine, (+)-N8-norphenserine and (+)-N1,N8-bisnorphenserine (B). Inhibitors of PKC (GF109203X, 2.5 µM), MEK1/2 (U0126, 5 µM) or MEK1 (PD98059, 10 µM) were added to the cells 30 min before adding (+)-phenserine (C). After 24 hr, cell proliferation was determined by MTS assay. *P<0.05, **p<0.01, and ***p<0.001 compared to control samples without drug, #*p*<0.05 compared to samples with (+)-phenserine only (one-way ANOVA followed by Bonferronís multiple comparison post hoc test). Results are expressed as mean ± SEM. Chemical structures of (−)- and (+)-phenserine, (+)-N1-norphenserine, (+)-N8-norphenserine and (+)-N1,N8-bisnorphenserine are shown in (D).

The SH-SY5Y human neuroblastoma cell line is widely used in the study of neurotrophic and neuroprotective mechanisms of centrally active drugs as these cells express a neuronal phenotype and possess a number of endogenous neuronal receptors [30], [31]. To establish whether (+)-phenserine induces neurotrophic effects in SH-SY5Y cultures, cells were incubated with increasing concentrations of compound and viability was assessed by quantifying cell proliferation by MTS assays. As illustrated in Figure 1A, a dose-dependent elevation in cell proliferation was observed after (+)-phenserine treatment (3 to 300 µM), which did not appear to be saturable and rose to 183% of control values. To define whether this action was either enantio-selective or compound specific, parallel cultures of SH-SY5Y cells were challenged with (−)-phenserine or with the three primary metabolites of (+)-phenserine at a well tolerated submaximal concentration (30 µM). As shown in Figure 1B, (−)-phenserine and (+)-N1-norphenserine induced an alike response to equimolar (+)-phenserine; however, the metabolites (+)-N8-norphenserine and (+)-N1,N8-bisnorphenserine lacked action on cellular proliferation. These metabolites differ from the primary drug by the replacement of methyl moieties by hydrogens in their pyrrolidine rings (Fig. 1D).

Determination of which signaling pathways are essential for (+)-phenserine-mediated neurotrophic actions was undertaken by utilizing specific pathway inhibitors. As illustrated in Figure 1C, the PKC inhibitor GF109203X and the MEK inhibitors U0126 (MEK1/2) and PD98059 (MEK1) fully attenuated (+)-phenserine mediated neurotrophic effects; suggesting that both PKC and MAPK pathways play physiological roles in (+)-phenserine induced neural cell proliferation.

### Neurotrophic Actions of (+)-phenserine are Maintained in SH-SY5Y Cells Expressing APP_SWE_ and in the Presence of Aβ and Oxidative Stress

**Figure 2:**
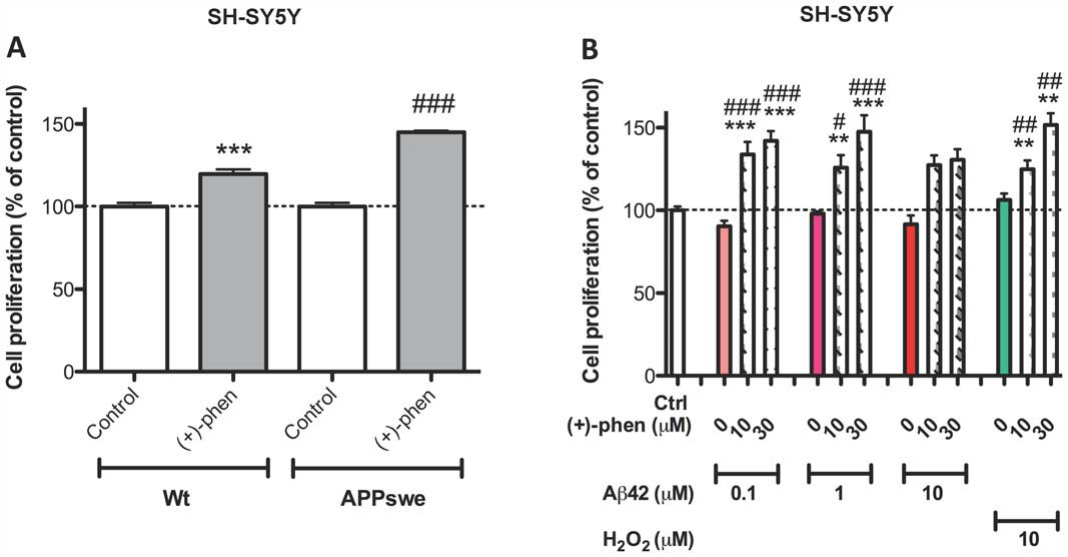
Neurotrophic actions retained in APPswe transfected and wt SH-SY5Y cells challenged with Aβ42 and H_2_O_2_. Cells were exposed to (+)-phenserine (30 µM) for 24 hr before measuring cell proliferation using MTS assay in (A). 10 or 30 µM (+)-phenserine was added 24 hr before adding 0.1, 1 or 10 µM Aβ42 or 10 µM H_2_O_2_ before measuring cell proliferation in untransfected SH-SY5Y cells (B). **p<0.01, and ***p<0.001 compared to control samples without drug, ##*p*<0.01 and ###*p<*0.001 compared to samples with (+)-phenserine only (one-way ANOVA followed by Bonferronís multiple comparison post hoc test). Results are expressed as mean ± SEM.

To establish whether (+)-phenserine would induce neurotrophic actions in SH-SY5Y cells stably transfected with the APP_SWE_ variant linked to familial AD, the cells were challenged with (+)-phenserine alongside wt cells. As illustrated in Figure 2A, 30 µM (+)-phenserine readily induced an elevation in cell viability in both cell types and, indeed, evoked a significantly greater effect in cells expressing APP_SWE_ (145% of control values versus 120% for wt cells). In light of heightened levels of Aβ and oxidative stress in the AD brain, wt SH-SY5Y cells were challenged with (+)-phenserine in the presence and absence of elevated but subtoxic concentrations of Aβ_42_ to elucidate whether neurotrophic actions would persist under conditions mimicking an AD setting. As shown in Figure 2B, a (+)-phenserine-induced increase in cellular proliferation endured in the presence of 0.1 and 1.0 µM aged Aβ_42_ and demonstrated a strong trend toward elevation in the presence of 10 µM Aβ_42_. As oxidative stress has been implicated in Aβ_42_-induced neuronal dysfunction [32], the neurotrophic actions of (+)-phenserine were, likewise, characterized in the presence of a sub-lethal H_2_O_2_-induced oxidative insult (H_2_O_2_ 10 µM) and, likewise, were retained (Fig. 2B).

### Neuroprotective Actions of (+)- and (−)-phenserine in SH-SY5Y Cell Cultures

**Figure 3:**
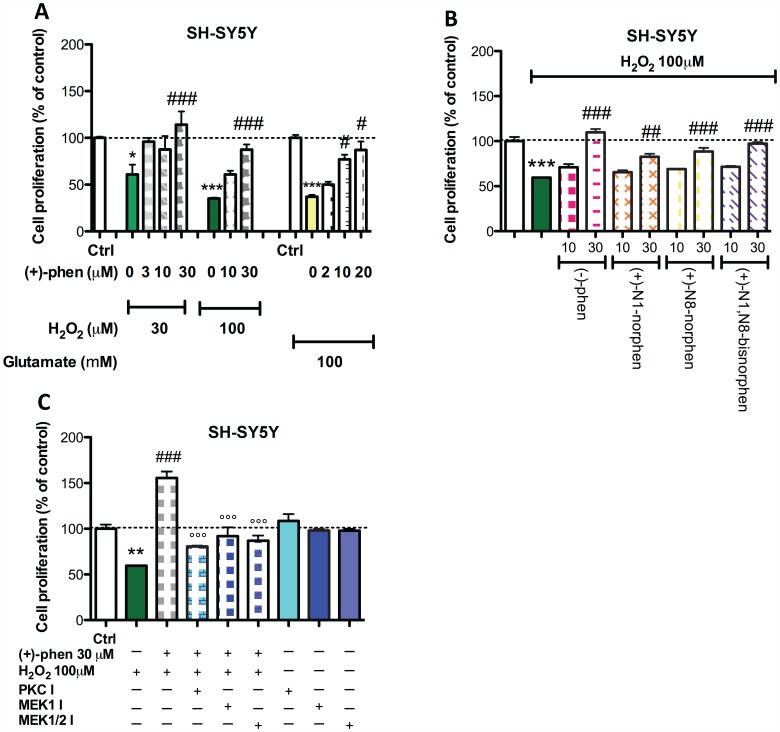
Phenserine and primary metabolites exert neuroprotective actions against H_2_O_2_ and glutamate-induced toxicity in SH-SY5Y cells. Cells were exposed to 3, 10 or 30 µM (+)-phenserine 24 hr before addition of 30–100 µM H_2_O_2_ or to 100 mM glutamate in (A), or to (−)-phenserine, (+)-N1-norphenserine, (+)-N8-norphenserine or (+)-N1,N8-bisnorphenserine 24 hr before addition of 100 µM H_2_O_2_ in (B). In (C), inhibitors of PKC (GF109203X, 2.5 µM), MEK1/2 (U0126, 5 µM) or MEK1 (PD98059, 10 µM) were added to the cells 30 min before adding 30 µM (+)-phenserine. 100 µM H_2_O_2_ was added after 24 hr and after additional 24 hr incubation, cell proliferation was determined by MTS assay. *P<0.05 **p<0.01, and ***p<0.001 compared to control samples without drug, #p<0.05, ##*p*<0.01 and ###*p<*0.001 compared to samples with (+)-phenserine only, °°° compared to samples exposed to (+)-phenserine and H_2_O_2_ (one-way ANOVA followed by Bonferronís multiple comparison post hoc test). Results are expressed as mean ± SEM.

To examine whether stimulation with (+)-phenserine could protect SH-SY5Y cells from toxic levels of H_2_O_2_ induced oxidative stress, cells were incubated with increasing concentrations of (+)-phenserine (3 to 30 µM) during exposure to a toxic oxidative stress challenge (Fig. 3). A significant H_2_O_2_-induced decline in cell viability was evident at 30 and 100 µM concentrations (Fig. 3A). (+)-Phenserine provided concentration-dependent protection against H_2_O_2_ induced cell death, providing full amelioration at a dose of 3 uM during 30 uM H_2_O_2_ challenge (Fig. 3A) but requiring greater concentrations on encounter with 100 µM H_2_O_2_. As illustrated in Figure 3B, equimolar concentrations of (−)-phenserine as well as each of (+)-phenserine’s three primary metabolites afforded similar neuroprotective activity.

To characterize this protective response, selective biochemical pathway inhibitors were added in parallel studies and, as shown in Figure 3C, suggest that this (+)-phenserine action involved PKC and MEK1/2 mediated cascades.

### (+)-Phenserine is Neurotrophic to Cultured Neural Progenitor Cells

**Figure 4:**
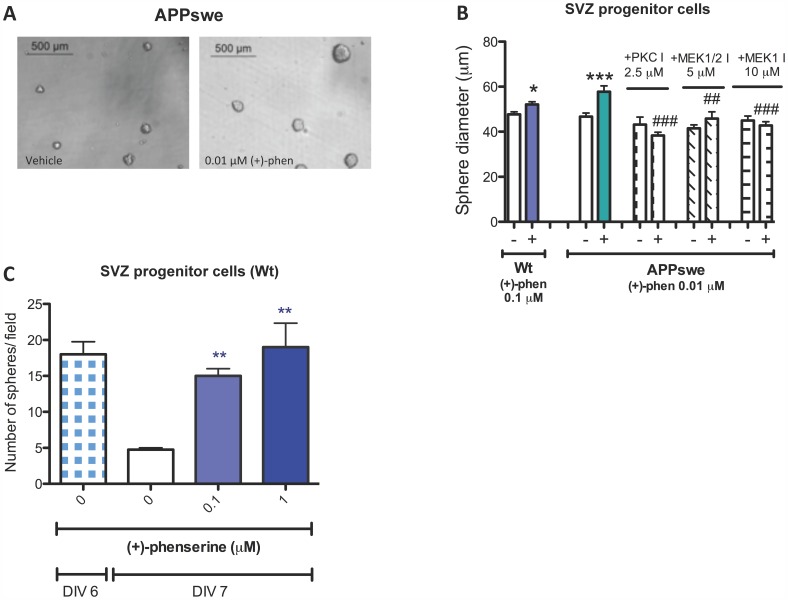
Enhanced subventricular progenitor cell survival and increased size of Tg2576 mouse neurospheres after (+)-phenserine exposure. Representative images show SVZ progenitor cells exposed to vehicle or 0.01 µM (+)-phenserine (A). Effects of the MEK1/2 inhibitor U0126, MEK1 inhibitor PD98059, and PKC inhibitor GF109203X on neurosphere size and neurosphere density in the presence of (+)-phenserine are shown in (B). ***P<0.001 compared to control samples without (+)-phenserine or inhibitor, ##*P*<0.01 and ###*P<*0.001 compared to samples with (+)-phenserine only (one-way ANOVA followed by Bonferronís multiple comparison post hoc test). The number of neurospheres composed of SVZ progenitor cells, isolated from the lateral (LGE) and medial (MGE) ganglionic eminence at embryonic day 13.5, were measured 7 days after exposure to 0.05, 0.1 and 1 µM (+)-phenserine (C). *p<0.05, **p<0.01, and ***p<0.001 compared to samples without (+)-phenserine. Data were analysed with one-way ANOVA followed by Dunnetts post hoc test. Results are expressed as mean ± SEM.

Key vulnerable proliferative cell populations within the brain are neural progenitor cells localized within the subventricular zone (SVZ) and the subgranular zone of the hippocampus that give rise to the genesis of new neurons within the adult brain [33]. As a consequence, SVZ cells from rodent embryos were isolated and cultured in epidermal growth factor-containing media to support their generation of neurospheres. The addition of pharmacologically relevant concentrations of (+)-phenserine (0.01 or 0.1 µM) demonstrated a trend to increase the number of neurospheres (not shown) and significantly enlarged their size (Fig. 4A,B), whether derived from wt mice or from mice that overexpress human APP with the Swedish double mutation (Tg2576). As before, by utilizing selective pathway inhibitors (+)-phenserine induced neurotrophic actions on SVZ progenitor cells were determined to involve PKC and MEK1 −1/2 signaling (Fig. 4B).

One day following DIV6, a significant time-dependent degeneration and loss of neurospheres was evident. These declined from 18 neurospheres/field on DIV6 to 5 neurospheres/field on DIV7 (Fig. 4C). Treatment with (+)-phenserine substantially slowed this neurosphere loss, with a significant difference in the remaining neurosphere number being observed between those treated with (+)-phenserine and vehicle on DIV7 (Fig. 4C).

### (+)-Phenserine Exerts Neurotrophic Actions in vivo in both wt and AD Tg2576 mice

**Figure 5:**
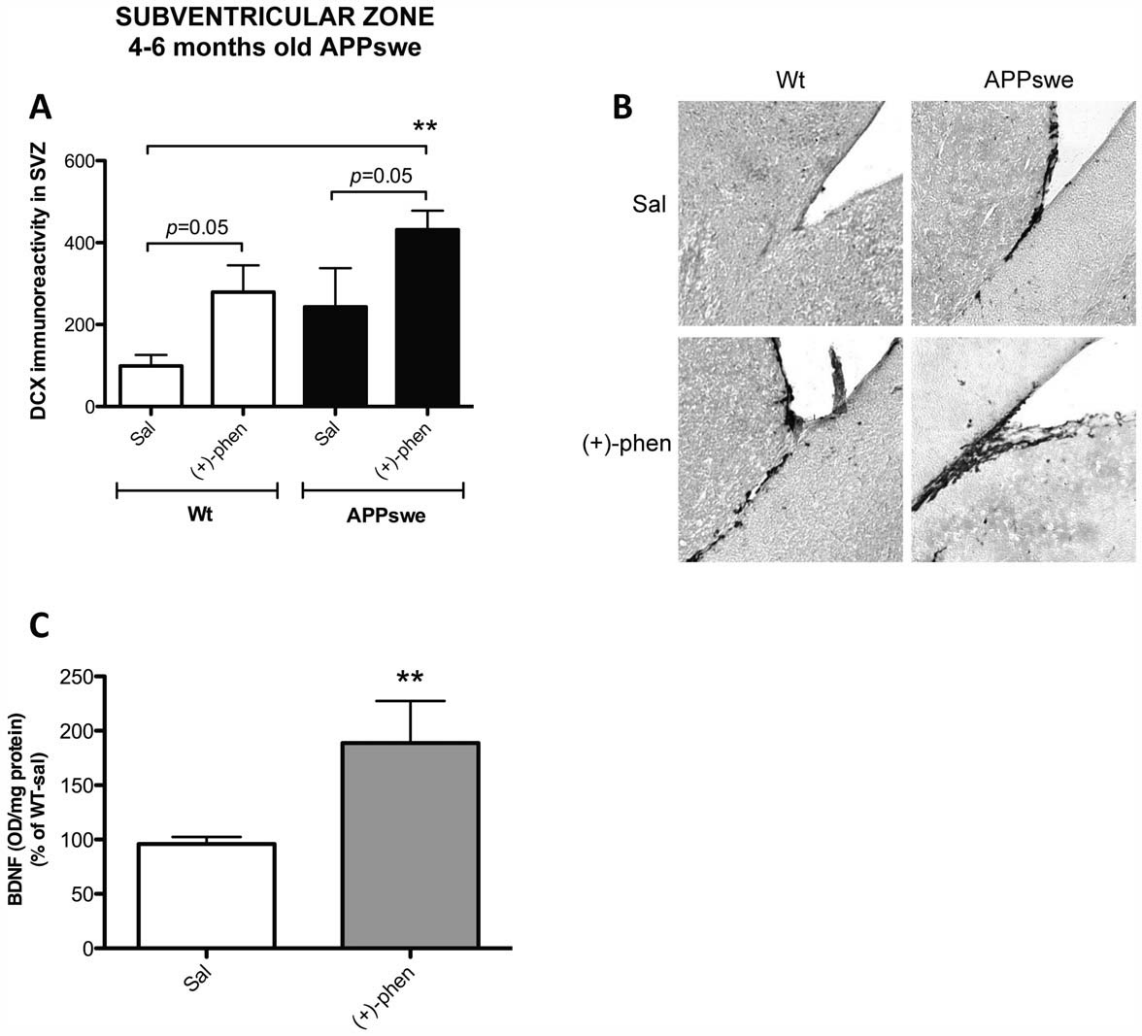
Enhanced doublecortin reactivity in the SVZ of 4–6 months old APPswe mice after (+)-phenserine treatment. Doublecortin (DCX) immunoreactivity in the subventricular zone (SVZ) in 4–6 months old mice are shown in A and B. Cortical BDNF levels in wt 4–6 months old mice are shown in C. **p<0.01 compared to wt mice treated with saline. Results are expressed as mean ± SEM.

To elucidate whether (+)-phenserine-induced neurotrophic actions on SVZ derived neural progenitor cells that were evident in cell culture translate *in vivo*, adult wt and Tg2576 mice were treated with (+)-phenserine (25 mg/kg) for 16 consecutive days. DCX is a microtubule-associated protein that is expressed within a limited phase during the development of neuroblasts in both the developing and adult brain [34]. Its expression is hence used as a reliable marker of neurogenesis [35], [36]. Systemic administration of (+)-phenserine significantly increased the DCX immunoreactivity within the SVZ of both wt and AD Tg2576 mice by two- to three-fold (Fig. 5A,B).

To assess the actions of (+)-phenserine on neurotrophic factors, the levels of BDNF were measured in the cerebral cortex of drug and saline treated wt mice. BDNF levels were elevated by 97% in (+)-phenserine treated mice compared to saline controls (p<0.01, Fig. 5C).

## Discussion

Substantial evidence indicates that toxic APP metabolites and, in particular, Aβ induce oxidative stress and diminishing neurotrophic factors that lead to neuronal dysfunction and demise, and a decline in neurogenesis in the AD brain. In light of this, well-tolerated agents that mitigate these effects may impact disease progression if provided sufficiently early during the disease course. In this regard, within the current study we assessed the actions of the clinical drug candidates (+)- and (−)-phenserine as the former has recently been reported to lower CSF levels of APP metabolites, Aβ, tau and inflammatory markers in subjects with mild cognitive impairment [20], as well as to augment neurogenesis following progenitor cell transplantation in AD APP23 transgenic mice [26]. Herein, we demonstrated that (+)-phenserine exerted neurotrophic and neuroprotective effects in human neuroblastoma SH-SY5Y cells as well as in mouse primary SVZ neural progenitor cells in culture. These actions translated into 4–6 month old Tg2576 mice in which (+)-phenserine administration increased the expression of the early neuronal marker DCX within the SVZ, one of the two major neurogenic zones.

The few existing studies to date have reported that neurogenesis persists in the adult human brain and although neuronal loss is persistent in AD, altered hippocampal neurogenesis has been found in studies on autopsy brain from AD patients [37], [38]. A diminished capacity for neurogenesis in AD brain might be a consequence of reduced levels of growth factors and neurotrophic signaling, as well as increased accumulation of Aβ oligomers and fibrils during the progression of disease [39]. BDNF plays a pivotal role in neuronal development and for synaptic plasticity [40], [41], and levels of BDNF are decreased in the entorhinal cortex and the hippocampus in the AD brain [12], [42], [43]. Previous studies indicate that BDNF exerts neuroprotective effects in rodents and primates, and suggests a therapeutical value of elevating BDNF levels in AD [44]. BDNF activates intracellular signaling pathways that involve mitogen-activated protein kinases (MAPK/ERK), which are important for cell growth and differentiation. These pathways activate downstream transcription factor CREB, which in turn induces the expression of BDNF through a positive transcriptional feedback loop [40], [45]. A recent study identified APP as an important link in neuronal degeneration, and that deprivation of growth factors such as BDNF triggers the cleavage of APP by β-secretase to further activate caspases and to induce degeneration [46].

We propose that neurogenesis may provide a natural defense strategy against neurodegeneration in AD and, therefore, stimulating regenerative processes and cell survival in the brain may be clinically beneficial as a novel therapeutic strategy for AD. Increased neuroregeneration in the brain may be achieved by stimulating the brain’s endogenous stem cells in the major neurogenic zones, the SVZ in the lateral ventricles or in the dentate gyrus (DG) of the hippocampus. In support of this, it has recently been described that newborn mouse hippocampal granule cells receive input from circuits within the hippocampus as well as from distant cortical neurons, and that these newborn cells are functional and of importance for memory formation [47]. Studies to date have reported that drugs in clinical use for symptomatic treatment in AD have shown potential to stimulate neurogenesis. These include the acetylcholinesterase inhibitors (AChEIs) tacrine, galantamine, and the NMDA receptor antagonist memantine, which have been shown to increase neurogenesis in cortical neurons in culture and in mice [48]. However, the mechanisms via which these drugs potentially mediate neurotrophic effects remains largely unknown, as does their translational relevance.

The aim of the current study was to characterize potential neurotrophic/neuroprotective actions of (+)- and (−)-phenserine, to assess whether these were retained by their primary metabolites, (+)-N1-norphenserine, (+)-N8-norphenserine and (+)-N1,N8-bisnorphenserine, and investigate primary signaling pathways responsible for mediating these effects. Recent clinical trials have quantified both systemic and central time-dependent concentrations of (+)-phenserine and metabolites and suggest that a brain concentration of 3.5 µM was reached following a well tolerated oral 60 mg dose [20].

In cellular studies, a dose-dependent increase in cell proliferation was observed in SH-SY5Y cells after (+)-phenserine exposure. Neurotrophic effects were similarly observed by (−)-phenserine and by (+)-N1-norphenserine, but not by (+)-N8-norphenserine and (+)-N1,N8-bisnorphenserine, and, as demonstrated with (+)-phenserine, was sustained in cells bearing the APP_SWE_ mutation (Fig. 2A). However, as each of these compounds has been demonstrated to reduce APP levels in neuronal cells through translational inhibition of the APP mRNA 5′UTR region [23], [24], one can postulate that these effects are likely mediated through alternate mechanisms. In this regard, the increased cell proliferation observed in SH-SY5Y cells after (+)-phenserine exposure appeared to be mediated in large part through PKC and MEK dependent pathways (Fig. 1C). Cellular proliferation and cell cycle arrest is tightly regulated by MEK1 and MEK2, where MEK2 has been suggested to play a particularly important role in promoting cell survival [49], [50]. PKC acts upstream from ERK and is thus indirectly involved in the MAPK-CREB signaling pathway [51].

In addition to Aβ, oxidative stress mediated by reactive oxygen species, such as H_2_O_2_, and glutamate-induced excitotoxicity promote neuronal degeneration and are suggested to play important roles in AD [52], [53]. Our challenge of SH-SY5Y cells with sub-lethal concentrations of Aβ and H_2_O_2_ demonstrated that the neurotrophic action of (+)-phenserine was sustained in a milieu bearing characteristics common to the AD brain. At higher concentrations of H_2_O_2_ that induced a reduction in cellular viability, (+)-phenserine exerted neuroprotective actions in a dose-dependent manner (Fig. 3A). Similarly, (−)-phenserine proved capable of mitigating glutamate-induced toxicity, and this translated to primary hippocampal neurons (Fig. S1). Such neuroprotective actions were, likewise, mediated in large part via PKC- and MEK/ERK- dependent pathways.

In a second *in vitro* model system, we used SVZ progenitor cells derived from wt and Tg2576 mice to study effects on both growth and survival. Similar to our findings in SH-SY5Y cells, but importantly at concentrations (0.1–1.0 µM (Fig. 4A–C)) that are readily achievable in both preclinical animal models and humans [20], an increase in cell proliferation was observed as increased size of neurospheres in culture, which was shown to be mediated largely through PKC and MEK dependent pathways. In addition, in a dose-dependent manner, (+)-phenserine enhanced the survival of neurospheres that routinely undergo degeneration by DIV7, in a manner similar to primary cerebrocortical cultures [54].

To elucidate whether the neurotrophic actions observed in vitro translated into a relevant *in vivo* model system, we investigated the effects of (+)-phenserine treatment in Tg2576 mice overexpressing human APP with the Swedish double mutation (Tg2576). These mice possess soluble oligomeric Aβ assemblies at young age whereas Aβ plaques are formed at later ages [55], [56]. The early neuronal marker DCX, which plays an important role in neuroblast migration, as well as BDNF that is implicated in cell proliferation, neuronal differentiation and in the integration of neurons, were selected markers for assessing effects on neurogenesis in these animals. Within the SVZ of the lateral ventricle, a region that together with the dentate gyrus constitutes a major site for adult neurogenesis, cells positive for DCX were shown along the rostral migratory stream to the olfactory bulb and developed neurite outgrowths after leaving the SVZ. (+)-Phenserine not only increased the cell proliferation of SVZ progenitor cells in culture but also induced the expression of DCX within the SVZ of pre-plaque, 4–6 months old Tg2576 mice. A trend towards an elevation in DCX immunoreactivity was observed in the Tg2576 mice compared to wt mice. This may reflect an increased neurogenesis, in line with a few previous studies in postmortem AD brain showing an elevated number of neural progenitor cells compared to healthy controls [37], [38], [57]. An increased neurogenesis may then reflect the brain’s own defence capacity to compensate for neuronal dysfunction and synaptic impairment caused by Aβ or other pathological abberations in the brain. A similar trend to an increased DCX immunoreactivity by (+)-phenserine was evident in treated wt mice, and quantification of BDNF levels within the cerebral cortex demonstrated a (+)-phenserine-induced up to a 2-fold rise (Fig. 5C), suggesting that the agent has actions on neurotrophic processes as well as on neurotrophin expression within the brain. In further studies, this elevation in BDNF levels was evident following (−)-phenserine (7.5 mg/kg i.p. daily) in AD Tg mice (elevating hippocampal BDNF from 0.0865 to 0.101 pg/ug protein, p<0.05).

We have earlier demonstrated that treatment of APP23 Tg mice with (+)-phenserine not only augmented the survival of transplanted neural progenitor cells within the brain but favored the *in vivo* differentiation of these cells towards expression of a neuronal rather than a glial phenotype [26]. In a more recent study in alike 4–6 months old Tg2576 mice, a significant elevation in BrdU-positive proliferating cell number as well as an increase in the number of DCX positive cells with extending dendrites were evident within the hippocampus of (+)-phenserine treated mice, in line with the DCX increase found in our present study. Importantly, a significant reduction in Aβ_42_ levels and a rise in synaptophysin expression were evident in the cerebral cortex of these mice [personal communication with Amelia Marutle, Karolinska Institutet, Stockholm, Sweden]. A number of studies imply that BDNF plays an important role in the survival of newborn hippocampal neurons, dendritic growth and synaptogenesis, and that these processes involve signaling pathways that include MAPK and PKC [40], [58]. Hence, this neurotrophin may link together several of the effects presented herein.

In conclusion, we report that (+)-phenserine exerts actions fundamental to neuronal maintenance and regeneration; providing neurotrophic/protective support to impact cell proliferation and survival *in vitro* in an environment with characteristics common to AD as well as *in vivo* in a key neurogenic zone within the brain of mice. Our data implicate involvement of the MAPK signaling pathway including enhancement of BDNF levels. Receptor binding studies to investigate potential molecular targets thus far have revealed that (+)-phenserine does not directly bind to the most prevalent neurotransmitter receptors in the brain, including neuronal nicotinic acetylcholine receptors, muscarinic, dopaminergic, histamine or to 5-HT receptors [59]. Hence, further studies are warranted to elucidate the key mechanisms underpinning the effects presented herein. The present studies support the concept that (+)-phenserine possesses neurotrophic and neuroprotective actions in addition to its amyloid lowering properties, and further studies will evaluate the functional and cognitive impact of these effects in AD patients.

## Supporting Information

Figure S1 **Phenserine ameliorates glutamate mediated toxicity in primary hippocamal neurons.** Cells were exposed to glutamate (50 µM) in the presence and absence of (−)-phenserine (5 µM). **p<0.01 compared to samples without (−)-phenserine, ##*p*<0.01 compared to samples with (−)-phenserine only. Data were analysed with one-way ANOVA followed by Dunnetts post hoc test. Results are expressed as mean ± SEM.
(DOCX)
