# Supplementary material for: Neurotrophic and Neuroprotective Actions of (−)- and (+)-Phenserine, Candidate Drugs for Alzheimer’s Disease
Source: PLoS One. 2013 Jan 30;8(1):e54887. doi: 10.1371/journal.pone.0054887 (PMC3559887; doi:10.1371/journal.pone.0054887)
Supplement: Figure S1 — Phenserine ameliorates glutamate mediated toxicity in primary hippocamal neurons. Cells were exposed to glutamate (50 µM) in the presence and absence of (−)-phenserine (5 µM). **p<0.01 compared to samples without (−)-phenserine, ##p<0.01 compared to samples with (−)-phenserine only. Data were analysed with one-way ANOVA followed by Dunnetts post hoc test. Results are expressed as mean ± SEM. (DOCX) [file pone.0054887.s001.docx]

**Supporting information**

Cultured hippocampal neurons in triplicate on DIV 7 were treated with (-)-phenserine (5 μM) followed by the addition of an excitotoxic concentration of glutamate (50 μM).  Neuronal viability was assessed 24h after addition of glutamate. The results are plotted as percent neuronal survival ± SD. As shown in Fig. S1, glutamate significantly reduced cell viability by 53.5%, which was mitigated by (-)-phenserine (73.5% of control levels).

**Materials and Methods** Hippocampal cultures were prepared from embryonic day eighteen to twenty (E18-20) Sprague Dawley embryos as described previously [1,2].  Specifically, the hippocampi were dissected and placed into ice-cold Hanks solution with 1mM HEPES and 1μl/ml penicillin-streptomycin (10,000 units penicillin and 10,000μg/ml streptomycin).  The tissues were trypsinized with 1.25% trypsin (50μl/ml of Gibco 2.5% 10x Trypsin) in 10 ml Hanks and incubated at 37°C for 15 min, and then rinsed in 10 ml Hanks.  Trypsinization was inhibited with soybean trypsin Inhibitor (2mg/ml) in 10ml Hanks and incubated at 37°C for 5 min.  Tissues were then washed (10 ml of Gibco Minimum Essential Medium (MEM) containing in mM:  10.0 sodium bicarbonate, 1.0 pyruvate, 20.0 potassium chloride, 1.0 HEPES, 10% fetal bovine serum, and 2.0 L-glutamine, pH 7.2), and diluted with MEM to achieve a seeding density of 250,000 cells/ml into dishes coated with poly-D-lysine (50 μg/ml).  The hippocampal cells were incubated for 4 hr at 37°C in a humidified incubator containing 95% air/5% CO2.  Thereafter, cell media was aspirated and replaced with Neuralbasal media (containing 2% B27 supplement, 1M HEPES, 2mM L-glutamine, and 50mg/ml gentamicin) that was preheated to 37°C and added in the same volumes as the plating volumes of MEM. On day in vitro (DIV) 3 and every other day thereafter, one-fifth of the culture medium was removed and replaced with fresh media to replenish vital nutrients. Cultures were then used in experiments on DIV 7.

**
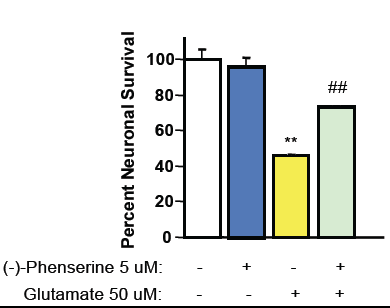
**

1. Cheng B, Furukawa K, O'Keefe JA, Goodman Y, Kihiko M, et al. (1995) Basic fibroblast growth factor selectively increases AMPA-receptor subunit GluR1 protein level and differentially modulates Ca2+ responses to AMPA and NMDA in hippocampal neurons. J Neurochem 65: 2525-2536.

2. Jiang X, Tian F, Mearow K, Okagaki P, Lipsky RH, et al. (2005) The excitoprotective effect of N-methyl-D-aspartate receptors is mediated by a brain-derived neurotrophic factor autocrine loop in cultured hippocampal neurons. J Neurochem 94: 713-722.
